# Computational Oncology

**Published:** 2018-03-30

**Authors:** RK Perez, R Kang, R Chen, JG Castellanos, AR Milewski, AR Perez

**Affiliations:** 1 University of California, San Francisco, 505 Parnassus Ave, San Francisco, CA 94143, USA; 2 Weill Cornell Medicine, 1300 York Ave, New York City, NY 10021, USA

## Letter to Editor

Medicine is quickly moving towards personalization. In oncology, patient tumors are now frequently sequenced to determine the mutations driving their cancer. Mutational knowledge directly informs oncologists about potential targeted therapies and prognoses. In melanoma, if a patient has the V600E mutation, they are a candidate for vemurafenib treatment, a targeted therapy shown to have success in battling late stage melanoma [1]. Paradoxically, however, if the patient does not have the V600E mutation then vemurafenib treatment can promote tumor growth [2]. Making the correct call regarding the patient’s mutational status is essential to providing the patient with the best clinical care. The sequencing of patient tumors to identify mutations that are targetable with pharmacotherapy represents a pillar of precision medicine. This foundation depends critically on bioinformatics and computation. The sequencing of tumor genomes, their alignment to a reference genome, and their associated mutation calling or differential expression analysis are all bioinformatics methods that inform oncologists about the genetic makeup of a patient’s cancer. Armed with this knowledge, the oncologist can individualize therapy based on a patient’s individual cancer.

**Figure 1:**
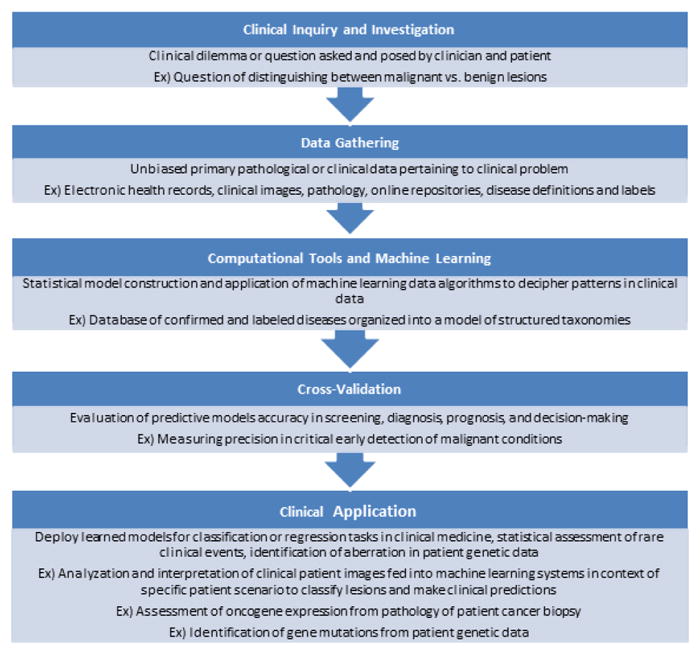
Application of data integration through machine learning to clinical scenarios presented by patients and physicians involved in precision medicine.

Computational tools are quickly incorporating themselves into the forefront of oncology. The introduction of machine learning models to diagnose cancer from clinical imaging or pathology slides is one such incorporation that promises to revolutionize how cancer is diagnosed. A recent report from scientists at Stanford University illustrated the power of machine learning’s ability to aid physicians in diagnosing skin cancer [3]. In their study, they employed a model, known as a deep convolutional neural network, which correctly classified various skin malignancies with accuracy similar to that of twenty-one board-certified dermatologists. Such a tool could represent a screening test that enhances a physician’s ability to diagnosis skin cancer by simply uploading an image of a lesion into the model and receiving a prediction on whether the lesion is likely to be malignant or not. Machine learning models are rapidly finding their way into all realms of oncology, from cancer diagnosis to cancer prognosis, and represent a powerful and upcoming tool in the field (Figure 1).

With the advent of precision medicine and biomedical machine learning, the tools needed to understand patient pathology have become more computational. Already, computational methods are being deployed in analyzing patient data in cancer and non-cancer settings, and advances in machine learning algorithms promise to assist physicians in all realms of medicine [4–6]. Nowhere has the adoption of bioinformatics into clinical medicine been more pronounced than in oncology where its potential to influence clinical decision-making is immense.

However, oncologists must understand the methods underlying tests that guide their clinical decision-making. Appreciating the limits of computational tools is as important as appreciating their potential. Knowing the quality of the data and parameters upon which bioinformatics software is developed is critical in determining whether a tool’s output is to be trusted. As oncology becomes more computational, the need for oncologists to have an understanding of the field’s computational methods increases. Basic instruction in bioinformatics should be a component of oncology training, so that oncologists not only understand the methods underlying their tools, but also are able to effectively utilize these methods in a way that advances both our understanding of cancer and improves patient care.
